# Identification of risk factors for post-induction hypotension in patients receiving 5-aminolevulinic acid: a single-center retrospective study

**DOI:** 10.1186/s40981-020-00340-9

**Published:** 2020-05-12

**Authors:** Tomoaki Yatabe, Takashi Karashima, Motohiko Kume, Yu Kawanishi, Hideo Fukuhara, Tetsuya Ueba, Keiji Inoue, Yoshiyasu Okuhara, Masataka Yokoyama

**Affiliations:** 1 Department of Anesthesiology and Intensive Care Medicine, Kochi Medical School, Kohasu, Oko-cho, Nankoku, Kochi 783-8505 Japan; 2 Department of Urology, Kochi Medical School, Kochi, Japan; 3 Medical safety management center, Kochi Medical School Hospital, Kochi, Japan; 4 Department of Neurosurgery, Kochi Medical School, Kochi, Japan; 5 Center of Medical Information Science, Kochi Medical School, Kochi, Japan

**Keywords:** 5-Aminolevulinic acid, Anesthesia, Hypotension

## Abstract

**Background:**

5-Aminolevulinic acid (5-ALA) is useful as a photodynamic agent, but its use commonly leads to hypotension. Although avoiding a mean arterial pressure (MAP) < 60 mmHg is important, the incidence of MAP < 60 mmHg when using 5-ALA is unclear. Therefore, we conducted a retrospective study to assess the incidence of post-induction hypotension and identified risk factors of this phenomenon.

**Methods:**

One-hundred and seventy-two consecutive patients who underwent transurethral resection of the bladder tumor or craniotomy with the use of 5-ALA were enrolled. The primary outcome was the incidence of post-induction hypotension, defined as MAP < 60 mmHg during the first 1 h after anesthesia induction. We divided participants into the normal blood pressure group (group N) and the hypotension group (group L).

**Results:**

The incidence of post-induction hypotension was 70% (group *L* = 121, group *N* = 51). Multivariate analysis revealed that female sex was an independent factor of post-induction hypotension (odds ratio [OR] 3.95; 95% confidence interval [CI] 1.21–12.97; *p* = 0.02). Systolic blood pressure < 100 mmHg before anesthesia induction and general anesthesia were also identified as significant independent factors (OR 13.30; 95% CI 1.17–151.0; *p* = 0.04 and OR 25.84; 95% CI 9.80–68.49; *p* < 0.001, respectively).

**Conclusions:**

The incidence of post-induction hypotension was 70% in patients using 5-ALA. Female sex, systolic blood pressure < 100 mmHg before anesthesia induction, and general anesthesia might be independent factors of post-induction hypotension when using 5-ALA.

## Background

5-Aminolevulinic acid (5-ALA) is a natural amino acid that is a precursor of protoporphyrin IX (Pp IX), which is a photosensitizer [1]. 5-ALA is used for photodynamic diagnosis of malignant disease, because exogenous administration of 5-ALA leads to the accumulation of Pp IX in cancer cells [2]. A recent meta-analysis of bladder cancer described that fluorescent cystoscopy using photodynamic agents, such as 5-ALA, was associated with a reduced risk of recurrence as compared with traditional cystoscopy [3]. Another meta-analysis showed a significant increase in the gross total resection rate with 5-ALA-guided surgical resection in high grade glioma [4].

On the other hand, a previous systematic review reported that the overall incidence of adverse effects of 5-ALA was 25.5% and that hypotension comprised 60% of these events [5]. Although the mechanism underlying 5-ALA-induced hypotension is unclear, our previous case report suggested that some patients with 5-ALA-induced hypotension might not respond to conventional therapy, such as volume administration and ephedrine [6]. Thus, it is important for surgeons and anesthesiologists to identify risk factors of 5-ALA-induced hypotension. A recent single-center retrospective study reported that transurethral resection of the bladder tumor (TUR-Bt) with 5-ALA was more likely to cause hypotension as compared with TUR-Bt without 5-ALA [7]. In that study, multivariate analysis revealed that general anesthesia and regular use of renin–angiotensin system inhibitors were independent risk factors for hypotension, which was defined as a lowest systolic blood pressure < 80 mmHg [7]. Another study reported that hypotensive events occurred in 11% of patients, with hypotension defined as a drop in mean arterial blood pressure (MAP) of ≥ 20 mmHg [8]. Their multivariate analysis revealed that use of antihypertensive drugs was an independent risk factor for 5-ALA-induced hypotension [8].

However, intraoperative hypotension lacks a clear definition [9]. The Perioperative Quality Initiative consensus statement described that intraoperative MAP < 60–70 mmHg is associated with myocardial injury, acute kidney injury, and death [9]. In addition, one third of all intraoperative hypotensive episodes occurred before surgical incision [9]. Therefore, we conducted a single-center retrospective study to assess the incidence of post-induction hypotension caused by 5-ALA and to identify risk factors of this event in TUR-Bt and craniotomy, using a definition of hypotension of MAP < 60 mmHg.

## Methods

### Study population

The study was carried out with the approval of the ethics committee of Kochi Medical School Hospital (No. 2019-062). Since this was a retrospective study, the need for obtaining informed consent from patients was waived. Study subjects were consecutive patients who underwent scheduled TUR-Bt or craniotomy with 5-ALA for photodynamic diagnosis from October 2013 through April 2019. We excluded patients who were aged < 20 years, those who were included in a clinical trial for obtaining pharmaceutical approval, or who refused the use of their data.

All patients received 5-ALA 20 mg/kg (Alaglio, Chugai Pharmaceutical, Tokyo, Japan, or Alabel Oral, Nobelpharma, Tokyo, Japan) orally. Individual anesthesiologists decided on the anesthetic procedure in accordance with the patient’s condition. After the patient was admitted to the operating room, standard monitoring, namely, electrocardiography, non-invasive blood pressure measurement, and pulse oximetry, was performed. An arterial catheter was inserted into the radial artery for continuous blood pressure monitoring, if individual anesthesiologists decided on this monitoring. Details of anesthesia and treatment for hypotension were decided by the individual anesthesiologists.

### Data and outcomes

The following data were collected from the electronic medical records and the electronic anesthesia records: age; sex; height; weight; American Society of Anesthesiologists physical status (ASA-PS) classification; comorbidities (heart disease, diabetes mellitus, liver dysfunction, hypertension); use of calcium blocker, renin–angiotensin system (RAS) inhibitors, and psychiatric drugs; preoperative estimated glomerular filtration rate (eGFR) and hematocrit; systolic blood pressure, MAP, and heart rate before 5-ALA intake and before anesthesia induction; detail of the anesthetic procedure; intraoperative minimum systolic and mean blood pressure and heart rate; and use of ephedrine, phenylephrine, dopamine, noradrinaline, and adrenaline. Heart disease was defined as atrial fibrillation (Af), paroxysmal Af, pacemaker implantation, moderate and severe valve disease, history of percutaneous coronary intervention or coronary artery bypass grafting, and history of heart failure. We defined the blood pressure and heart rate before anesthesia induction as those when entering the operating room.

**Fig 1:**
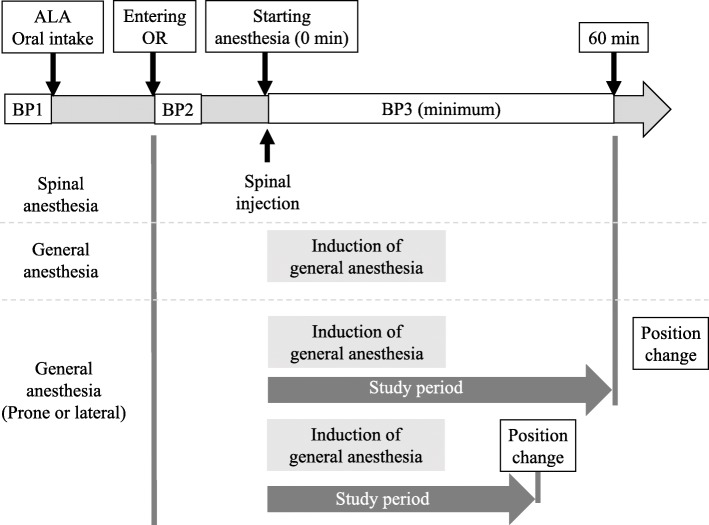
Definition of post-induction period and timing of blood pressure measurement. We defined post-induction period as the initial 60-min period taking place immediately after anesthesia induction. Starting anesthesia (0 min) was initiation of anesthesia induction. If a positional change from a supine to a prone or lateral recumbent position was performed during this 60 min in craniotomy, the study period was then defined as the time from anesthesia induction to just before the positional change. ALA, 5-aminolevulinic acid; OR, operating room; BP1, blood pressure before 5-ALA intake; BP2, blood pressure when entering operating room (before anesthesia induction); BP3, blood pressure during post-induction period

The primary outcome was the incidence of post-induction hypotension. We defined post-induction hypotension as follows: (1) MAP < 60 mmHg in accordance with a recent review article [9], (2) during the first 1 h after anesthesia induction, (3) anesthesia induction was defined as initiation of oxygenation in general anesthesia and spinal injection in spinal anesthesia, and (4) from anesthesia induction to just before positional change, if a positional change from a supine to a prone or lateral recumbent position was performed during the first hour in craniotomy (Fig. 1). Then, we divided the participants into two groups, namely, the normal blood pressure group (group N) and the hypotension group (group L).

### Statistical analysis

Data were expressed as median [interquartile range]. Statistical analysis was performed using the Wilcoxon rank-sum test and Fisher’s exact test, and logistic regression was performed using R version 3.3.3 (R Foundation for Statistical Computing, Vienna, Austria). A value of *p* < 0.05 was considered statistically significant. We included variables with *p* < 0.05 in univariate analysis in multivariate analysis.

## Results

A total of 172 patients were included in this study. Of these, 121 patients experienced post-induction hypotension (group L), and 51 patients did not (group N). Thus, the incidence of post-induction hypotension was 70%. The characteristics of these two groups are presented in Table 1. The proportions of females and ASA-PS = 3 in group L were significantly higher than those in group N (31 vs 14%, *p* = 0.02; 14 vs 2, *p* = 0.01, respectively). The proportion of patients who received TUR-Bt in group N was significantly higher than that in group L (82 vs 53%, *p* < 0.001). The proportion of heart disease as a comorbidity in group L was significantly higher than that in group N (17 vs 2%, *p* = 0.009). Although systolic and mean blood pressure before 5-ALA intake were similar, systolic blood pressure before anesthesia induction in group L was significantly lower than that in group N (122 [109, 136] vs 130 [117, 150] mmHg, *p* = 0.01). Difference in MAP before 5-ALA intake and before anesthesia induction ([MAP anesthesia induction]−[MAP before5-ALA intake]) in group L was significantly higher than that in group N (− 10 [− 3, 7] vs 2 [− 4, 11] mmHg, *p* = 0.01). The proportion of patients who received general anesthesia in group L was significantly higher than that in group N (88 vs 25%, *p* < 0.001).

**Table 1:** Patient background and pre-anesthesia data

		Group N (*N* = 51)	Group L (*N* = 121)	*P* value
Age	years	71 [64, 81]	74 [66, 81]	0.71
Female	*N* (%)	7 (14)	38 (31)	0.02*
Height	cm	162 [158, 168]	161 [155, 168]	0.20
Weight	kg	60 [52, 69]	59 [52, 69]	0.85
Body mass index	kg/m^2^	22.5 [20.2, 24.8]	22.8 [20.8, 25.1]	0.40
ASA-PS (1/2/3)	*N*	6/44/1	8/96/17	
ASA-PS = 3	*N* (%)	1(2)	17 (14)	0.01*
TUR-Bt	*N* (%)	42 (82)	64 (53)	< 0.001*
Preoperative condition				
Heart disease	*N* (%)	1 (2)	20 (17)	0.009*
Atrial fibrillation or PAf	*N* (%)	1 (2)	13 (11)	0.07
Other disease	*N* (%)	0 (0)	12 (10)	0.02*
Diabetes mellitus	*N* (%)	7 (14)	19 (16)	0.82
Liver dysfunction	*N* (%)	3 (6)	7 (6)	1.00
eGFR	ml/m^2^	73 [57, 84]	69 [57, 82]	0.64
Hypertension	*N* (%)	26 (51)	66 (55)	0.74
Use of calcium blocker	*N* (%)	21 (41)	52 (43)	0.87
Use of RAS inhibitor	*N* (%)	15 (29)	38 (31)	0.86
Use of psychiatric drugs	*N* (%)	1 (2)	5 (4)	0.67
Preoperative hematocrit	%	39.6 [37.7, 42.8]	38.5 [35.9, 42.0]	0.06
Pre ALA systolic BP	mmHg	128 [117, 143]	124 [114, 138]	0.17
Pre ALA mean BP	mmHg	92 [84, 101]	91 [84, 97]	0.41
Pre ALA heart rate	beats/min	65 [60, 74]	66 [60, 72]	0.98
ALA to anesthesia induction	min	167 [140, 202]	158 [142, 187]	0.51
Systolic BP before induction	mmHg	130 [117, 150]	122 [109, 136]	0.01*
Mean BP before induction	mmHg	92 [84, 102]	90 [84, 97]	0.41
Difference systolic BP	mmHg	4 [-7, 16]	-2 [-15, 11]	0.13
Difference mean BP	mmHg	2 [-4, 11]	-10 [-3, 7]	0.01*
Heart rate before induction	beats/min	65 [60, 74]	66 [60, 72]	0.98
General anesthesia	*N* (%)	13 (25)	107 (88)	<0.001*

*N* number, *ASA-PS* American Society of Anesthesiologists physical status classification, *TUR-Bt* transurethral resection of the bladder tumor, *PAf* paroxysmal Af, *eGFR* estimated glomerular filtration rate, *RAS* renin-angiotensin system, *ALA* 5-aminolevulinic acid, *BP* blood pressure, *Pre ALA* before 5-ALA intake, *ALA to anesthesia induction* time from 5-ALA intake to anesthesia induction, *before induction* before anesthesia induction, *Difference* difference in BP before 5-ALA intake and before anesthesia induction ([BP anesthesia induction]−[BP before5-ALA intake])

*p < 0.05

Details of the anesthesia procedure and treatment for hypotension are presented in Table 2. The anesthesia procedure in both groups was similar. Minimum systolic and mean blood pressure in group L were significantly lower than those in group N (68 [60, 75] vs 97 [86, 109] mmHg, *p* < 0.001; 49 [44, 55] vs 68 [63, 78] mmHg, *p* < 0.001, respectively). The minimum heart rate in group L was significantly lower than that in group N (68 [58, 79] vs 75 [68, 86] beats/min, *p* = 0.003). The proportions of use of ephedrine, phenylephrine, and noradrenaline in group L were significantly higher than those in group N (87 vs 49 %, *p* < 0.001; 38 vs 12 %, *p* < 0.001; 10 vs 0 %, *p* = 0.02, respectively).

**Table 2:** Detail of anesthesia procedure and treatment for hypotension

		Group N	Group L	*P* value
General anesthesia	*N* = 120	*N* = 13	*N* = 107	
Use of inhalation agent	*N* (%)	7 (54)	60 (56)	1.00
Dose of propofol	mg	100 [55, 110]	80 [60, 80]	0.34
Target concentration	μg/ml	3 [3, 3]	3 [3, 3.4]	0.33
Use of fentanyl	*N* (%)	8 (62)	65 (61)	1.00
Dose of fentanyl	μg	175 [100, 200]	100 [100, 200]	0.29
Use of remifentanil	N (%)	11 (85)	90 (84)	0.40
Dose of remifentanil	mg/hr	1.0 [0.4, 1.25]	0.6 [0.5, 1.00]	0.70
Spinal anesthesia	*N* = 52	N = 38	N = 14	
Use of hyperbaric bupivacaine	*N* (%)	35 (92)	12 (86)	0.60
Dose of bupivacaine	ml	2.5 [2.4, 2.8]	2.4 [2.2, 2.7]	0.51
Overall	*N* = 172	N = 51	N = 121	
Minimum systolic BP	mmHg	97 [86, 109]	68 [60, 75]	< 0.001*
Minimum mean BP	mmHg	68 [63, 78]	49 [44, 55]	< 0.001*
Minimum heart rate	beats/min	75 [68, 86]	68 [58, 79]	0.003*
Use of ephedrine	*N* (%)	25 (49)	105 (87)	< 0.001*
Dose of ephedrine	mg	8 [4, 12]	12 [8, 20]	< 0.001*
Use of phenylephrine	*N* (%)	6 (12)	46 (38)	< 0.001*
Dose of phenylephrine	mg	0.1 [0.1, 0.1]	0.3 [0.2, 0.5]	0.002*
Use of dopamine	*N* (%)	2 (4)	7 (6)	1.00
Use of noradrenaline	*N* (%)	0 (0)	12 (10)	0.02*
Use of adrenaline	*N* (%)	0 (0)	2 (2)	1.00

Dose of propofol, fentanyl, and remifentanil and target concentration of propofol for anesthetic induction were presented.

*N* number, *BP* blood pressure

*p < 0.05

We selected sex, ASA-PS, heart disease, decrease in MAP after ALA intake < 0, systolic blood pressure < 100 mmHg before anesthesia induction, and general anesthesia as variables for multivariate analysis. We excluded the surgical procedure (TUR-Bt or craniotmy) from this analysis, because this variable was strongly correlated with the selection of the anesthesia procedure (general anesthesia or spinal anesthesia). Thus, we included only the anesthesia procedure as a variable in multivariate analysis. This analysis revealed that female sex was an independent factor for post-induction hypotension in patients receiving 5-ALA (odds ratio [OR] 3.95; 95% confidence interval [CI] 1.21–12.97; *p* = 0.02). Systolic blood pressure < 100 mmHg before anesthesia induction and general anesthesia were also considered as independent factors that could affect post-induction blood pressure in patients receiving 5-ALA (OR 13.30; 95% CI 1.17–151.0; *p* = 0.04 and OR 25.84; 95% CI 9.80–68.49; *p* < 0.001, respectively) (Table 3).

**Table 3:** Multivariate analysis in normal and hypotension groups

*N* = 172	Reference	Odds ratio [95% CI]	*P* value
Gender (female)	Male	3.95 [1.21, 12.97]	0.02*
ASA-PS (ASA-PS = 3)	1 or 2	8.94 [0.68, 118.0]	0.10
Heart disease	No	10.40 [0.99, 110.0]	0.05
Systolic BP < 100 mmHg before induction	No	13.30 [1.17, 151.0]	0.04*
Decrease of mean blood pressure after ALA	No	1.26 [0.50, 3.19]	0.62
General anesthesia	Spinal	25.84 [9.80, 68.49]	< 0.001*

*N* number, *CI* confidence interval, *ASA-PS* American Society of Anesthesiologists physical status classification, *BP* blood pressure, *before induction* before anesthesia induction, *decrease of mean blood pressure after ALA* decrease of mean blood pressure after 5-aminolevulinic acid intake

*p < 0.05

## Discussion

We conducted a single-center retrospective study to assess the incidence of post-induction hypotension and to identify risk factors for this phenomenon in patients who received 5-ALA. Our data revealed that the incidence of post-induction hypotension, defined as MAP < 60 mmHg, was 70% in patients using 5-ALA. In addition, patients using 5-ALA required higher doses of ephedrine and phenylephrine, and noradrenaline was required in 10% of these patients. Multivariate analysis indicated that female sex, systolic blood pressure < 100 mmHg before anesthesia induction, and general anesthesia were independent factors for post-induction hypotension in patients receiving 5-ALA.

Several previous studies have reported 5-ALA-induced hypotension. Chung et al. described that hypotension occurred in 11% of glioma patients, when hypotension was defined as a drop in MAP of ≥ 20 mmHg within 3 h of 5-ALA administration, as compared to their MAP at 3 h before 5-ALA administration [8]. Bondad et al. defined hypotension as < 80% of the systolic or diastolic blood pressure before the ingestion of 5-ALA [10]. In this study, 83% and 88% of patients who underwent ureterorenoscopy procedures experienced hypotensive events within the 4-h period after ingestion of 5-ALA and within the 4 h after surgery [10]. Nohara et al. compared 109 patients who underwent TUR-Bt using 5-ALA and 83 patients who received conventional TUR-Bt, retrospectively [7]. In their study, systolic blood pressure before anesthesia and during anesthesia and surgery in the 5-ALA group was significantly lower than that in the conventional TUR-Bt group [7]. These studies focused on preoperative and postoperative pressure and on systolic blood pressure in particular. On the other hands, a recent review recommended that systolic blood pressure be maintained > 100 mmHg, and MAP be maintained at > 60–70 mmHg during anesthesia, because of the strong association between intraoperative hypotension and myocardial injury, kidney injury, and death [9]. In addition, one third of intraoperative hypotensive episodes occurred between anesthesia induction and surgical incision [9]. Thus, it is important to reveal the incidence and risk factors of MAP < 60 mmHg during this period in patients receiving 5-ALA. Then, our data showed that the incidence of post-induction hypotension was 70% in patients using 5-ALA overall, 89% in those receiving general anesthesia, and 27% in those receiving spinal anesthesia.

Our multivariate analysis suggested that female sex, systolic blood pressure < 100 mmHg before anesthesia induction, and general anesthesia were considered as independent factors that affect post-induction blood pressure. We set systolic blood pressure < 100 mmHg as a cutoff value in accordance with a recent review that recommended an intraoperative target value [9], since the safe threshold of preoperative blood pressure might not be established. In a previous study, general anesthesia and regular use of RAS inhibitors were associated with systolic blood pressure < 80 mmHg from anesthesia induction to surgery [7]. Another study showed that a history of hypertension and antihypertensive therapy were the only significant risk factors leading to hypotension after 5-ALA intake [8]. Although timing of hypotension is important when considering risk factors, general anesthesia is a risk factor of pre-incision hypotension because the anesthetic drugs per se cause this hypotension [9]. All patients who received RAS inhibitors stopped these agents on the operation day in our own and in previous [7] studies. In contrast to a previous study [7], regular use of RAS inhibitors was not considered to be a risk factor in our study. We could not draw a conclusion about the influence of antihypertensive therapy, because the difference in the definition of hypotension, dose and types of anesthetic agents, duration from the last intake of antihypertensive agents to anesthetic induction, preoperative infusion, and fluid intake differed across studies.

Sex was a risk factor only in our study. A previous review stated that sex should be taken into account as a predictive factor for the dosage of several drugs [11]. In fact, bioavailability via the oral route might be higher in females than in males [12]. Because sex differences in the plasma concentration of 5-ALA and Pp IX are unclear, further research on sex differences of this concentration might help to clarify this matter. Our study also showed that systolic blood pressure < 100 mmHg at anesthesia induction was one of the risk factors affecting post-induction blood pressure responses to 5-ALA. A previous study showed that the OR for systolic blood pressure at the time of anesthesia induction was 0.979 (95% CI 0.995–1.003, *p* = 0.08) about the systolic blood pressure less than 80 mmHg from anesthesia induction to the operation [7]. These results suggested that anesthesiologists should pay an attention to systolic blood pressure just before anesthesia induction.

This study has several limitations. First, our study was a single-center study. Thus, our results might not be generalizable to other hospitals, because we could not exclude that perioperative management, including the anesthesia method in our hospital that may have influenced these results. In particular, our hospital did not decide the protocol for treating intraoperative hypotension. Thus, detailed procedures depended on the attending anesthesiologists. For example, some anesthesiologists administered ephedrine or phenylephrine immediately after subarachnoid injection of local anesthetics to prevent hypotension. Second, although a previous animal study suggested the Pp IX led to arterial relaxation via cyclic guanosine monophosphate [13], the mechanism underlying 5-ALA-induced hypotension remains unclear [7]. Therefore, we could not determine whether there are synergistic effects between antihypertensive agents, inhalation agents, such as sevoflurane and desflurane, propofol, opioids, and 5-ALA. A previous small retrospective study reported decreased blood pressure in 53% of the participants within the 4-h period of observation after ingesting 5-ALA [10]. In our study also, 53% of patients experienced a decreased MAP after 5-ALA intake. Thus, 5-ALA itself might affect blood pressure. Further basic studies of the mechanism of 5-ALA-induced hypotension and large-scale prospective studies to reveal risk factors of this hypotension are required.

## Conclusion

Our study showed that incidence of post-induction hypotension was 70% in patients using 5-ALA during surgery. Female sex, systolic blood pressure < 100 mmHg before anesthesia induction, and general anesthesia might be independent factors that affect post-induction blood pressure in patients receiving 5-ALA. Future multi-center prospective studies are necessary to confirm our results.

## Data Availability

The datasets used and/or analyzed during the current study are available from the corresponding author on reasonable request.

## Abbreviations

5-ALA: 5-Aminolevulinic acid
Pp IX: Protoporphyrin IX
TUR-Bt: Transurethral resection of the bladder tumor
MAP: Mean arterial blood pressure
ASA-PS: American Society of Anesthesiologists physical status
RAS: Renin–angiotensin system
eGFR: Estimated glomerular filtration rate
Af: Atrial fibrillation
